# Relation between Lifestyle and Socio-Demographic Factors and Body Composition among the Elderly

**DOI:** 10.5539/gjhs.v8n8p172

**Published:** 2015-12-17

**Authors:** Fahime Zeinali, Nahal Habibi, Mehnoosh Samadi, Kamal Azam, Kurosh Djafarian

**Affiliations:** 1 Department of Clinical Nutrition, School of Nutritional Sciences and Dietetics, Tehran University of Medical Sciences, Tehran, Iran; 2 Department of Nutrition and Dietetics, Faculty of Medicine& Health Sciences, Universiti Putra Malaysia, Serdang, Malaysia; 3 Department of Nutrition, Paramedical School, Ahvaz Jundishapur University of Medical Sciences, Ahvaz, Iran; 4 Department of Epidemiology and Biostatistics, School of Public Health, Tehran University of Medical Science, Tehran, Iran

**Keywords:** body composition, socio-demographic factors, elderly, FMI, lifestyle, physical activity

## Abstract

**Background:**

Aging is accompanied by various changes that can cause changes in diet and body composition resulting sometimes in malnutrition and disability in the elderly. Changes in body composition among the elderly are mainly the result of physical inactivity and nutrition. This study was designed to examine the relationship between lifestyle and socio-demographic factors and body composition.

**Method:**

A cross-sectional study was carried out with 380 elderly people aged 60 and over in district 5 of Tehran, Iran. Their body composition was measured by Bioelectrical Impedance Analysis and the Actigraph device was used for assessing physical activity patterns. A three-day food recall was conducted to measure their intake of energy and macronutrients. Lifestyle and socio-demographic information were collected by interview using a pretested questionnaire.

**Results:**

Overweight, obesity and central obesity were more prevalent in women than in men (p<0.001). Moreover, 57.1% and 18.7% of participants had high and very high fat mass index, respectively. High fat mass index was seen in 47% of men and 37.5% of women who had normal body mass index (BMI). Meanwhile, age, gender, physical activity, energy intake, the percentage of energy from fat and protein, educational level, job, television watching time, smoking, chronic diseases, and taking medicine were significantly associated with anthropometric measurements (p<0.05).

**Conclusion:**

Overweight, obesity and high body fat percentage were common among the aged. Considering the factors that are significantly associated with body composition, programs that can increase their awareness about the dietary balance and suitable physical activity should be organized to address these problems.

## 1. Introduction

World health organization (WHO) called the present century as the century of the elderly and most problems that health systems in some developed countries are dealing with are related to this section of the community (Greengross, Murphy, Quam, Rochon, & Smith, 1997). The elderly is differently defined in different countries due to varying social and cultural conditions of each society. However, the point that every society around the world agrees on is the fact that the elderly still have many years ahead and must take care of themselves in order to have quality of life (Greengross et al., 1997). The United Nations (UN) estimated that globally the percentage of old people will increase from 10.5% in 2007 to about 21.8% by 2050 (The United Nations, 2006). The number of aged people in Iran is also increasing rapidly and Lang et al. (2010) estimated in 2010 that in 50 years they will comprise 20% of the Iran population (Lang et al., 2010).

Non-communicable diseases are the most common health problems among the aged causing disability and death. However, they are preventable if they adopted certain lifestyles (Hatami, Ardebil, Majlesi, Nozadi, & Parizadeh, 2004; McGough & Zumsteg, 2014). Aging comes with changes in the body composition, namely the decrease in fat free mass (FFM) and increase in fat mass (FM). The decrease in FFM generally happens due to the loss of skeletal muscle mass (sarcopenia). Sarcopenia reduces the Basal Metabolic Rate (BMR) and muscular power and consequently restricts the person’s functionality leading to disability (Campion, 1998; Doherty, 2003; Fielding et al., 2011; Visser & Harris, 2012). At the same time, the decrease in BMR and physical activity can cause obesity (Campion, 1998; St-Onge & Gallagher, 2010). Therefore, the elderly who suffer from sarcopenia and obesity (sarcopenic obesity) have higher risk of health problems (Baumgartner, 2000; Han, Tajar, & Lean, 2011). Sarcopenic obesity occurs with high prevalence of different metabolic illnesses including cardiovascular diseases (CVD), diabetes type 2, and cancer (Zamboni, Mazzali, Fantin, Rossi, & Di Francesco, 2008; Vainio & Bianchini, 2002). The U curve that shows the relationship between BMI and death indicates the independent and different effects of the lower FFM (the left side of the curve) and high FM (the right side of the curve) (Buffa, Floris, Putzu, & Marini, 2011). Various factors can affect body composition in the elderly with physical activity and diet being the most important (Yao et al., 2003). A study of Chinese adults showed that body fat mass was positively associated with consumption of restaurant foods and negatively associated with physical activity (Yao et al., 2003).

According to the literature, two studies have evaluated the association between the physical activity and diet, and body composition (Paeratakul, Popkin, Keyou, Adair, & Stevens, 1998; Popkin, Paeratakul, Zhai, & Ge, 1995). However, they used general methods of the measurement such as BMI to assess body fat. In addition, physical activity was determined by questionnaire. This study was designed to examine the body composition of the elderly in district 5 of Tehran, Iran and to determine its relationship with diet, physical activity, socio-demographic factors and lifestyle. The results of this study can be useful for improving the health of the aged people.

## 2. Materials and Methods

### 2.1 Study Population and Sampling Procedure

Health centers in Tehran are managed by the health department and indirectly by the municipality of Tehran. This cross-sectional study was carried out between February 2014 and August 2014 among old people who were under the purview of the health centers in district 5 of Tehran. Sample size formula by Green (1991) was used to determine the sample size of this study:

n= 50+8m (whereby “m” is equal to number of independent variables)

n= 50+8 (33) = 314 participants

An additional 20% from the calculated sample size was added making a total of 377 participants. A total of 380 aged (age 60 and above) were randomly selected (Hatami et al., 2004; Personal correspondence, 2001). Initially, a list of all the elderly in the health centers of district 5 was provided. Then, based on the ratio of population of each health center, 380 subjects aged 60 and over were selected using systematic sampling. Finally, these 380 subjects were contacted and invited to participate in the study. Inclusion criteria included willingness to participate in the study, age 60 years and over, ability to have unassisted activity, and not suffering from such illnesses cancer, stroke and heart attack. Those individuals who were not qualified to enter the study were replaced with others from the same health center. Those who fastened the Actigraph for less than 4 days or 10 hours per day were excluded.

Prior to the data collection, ethical clearance was obtained from the University Research Ethics Committee of Tehran University of Medical Sciences (Code_93-02-161-26402). In addition, written informed consent was obtained from the all participants.

### 2.2 Measures

The weight and body composition of the subjects were measured by body composition analyzer (TANITA, BC-418) made in Japan. To increase the precision of measurement, subjects were asked to keep normal hydration, not to eat or drink 4 hours prior to the test, to avoid drinking alcohol and caffeine and doing sports 12 hours before the test, and not to take diuretic medicine 7 days prior to the test. Where possible they were asked to urinate 30 minutes prior to taking the measurement. Fat Mass Index (FMI) was calculated by dividing the body fat mass in kilograms by the height squared in meters and categorized into low (men: ≤1.7 kg/m^2^, women: ≤ 3.8kg/m^2^), normal (men: 1.8-5.1 kg/m^2^, women: 3.9-8.1 kg/m^2^), high (men: 5.2-8.2 kg/m^2^, women: 8.2-11.7 kg/m^2^), and very high (men: ≥8.3 kg/m^2^, women: ≥11.8 kg/m^2^) (Kyle, Schutz, Dupertuis, & Pichard, 2003).

Subjects with BMI less than 20 kg/m^2^, between 20 kg/m^2^ and 24.99 kg/m^2^, between 25 kg/m^2^ and 29.99 kg/m^2^, and 30 kg/m^2^ or higher were classified as underweight, normal, overweight, and obese, respectively (National Institutes of Health, 1998). The waist circumference was measured using a stretch-resistant tape with 0.5 centimeters precision. Based on the Asian population cut-off, the waist circumference of more than 90 centimeters in men and more than 80 centimeters in women was considered as central obesity (WHO, 2004). The cut-off of National Institute Health (NIH) in 1998 has defined central obesity as waist circumference of more than 102 centimeters for men and more than 88 centimeters for women (National Institutes of Health, 1998).

In order to measure the physical activity an objective method using Actigraph GT3X accelerometer (Pensacola, Florida, USA) was implemented. The participants were asked to fasten the accelerometer to their waist for 7 days and record their sleeping hours. To calculate the physical activity the waking hours were extracted. Participants were asked to detach the Actigraph in bath and other water activities and report all the days and hours that they detached the device, for any reasons, to the researcher. To download the data from accelerometer and exporting them to Excel software, Actilife 4.4.0 (Pensacola) was used. Waking time counts were divided by the total waking time in minutes and count per minute (cpm) was calculated for every participant. The different levels of activity were defined according to Freedson’s cut-off points as inactivity (cpm≤100), light activity (cpm 101-1951), moderate activity (cpm 1952-5724), and vigorous activity (cpm>5724) (Freedson et al., 1998). In this study, moderate and vigorous levels of activity were combined together because vigorous activity is little among the elderly.

To determine the dietary intake 24-hour three-day food recall (two weekdays and one weekend) was used. The energy intake and the percentage of energy from macronutrients were extracted using Nutritionist 4 (four) (N4) software version 3.5.2 (N Squared Computing, San Bruno, CA, USA). The socio-demographic and lifestyle information was collected by interview using questionnaire which contained questions about age, gender, education, former job, current working status, television watching time, illnesses, taking medicine in the last 6 months, smoking, income, possessing home, marital status, ethnicity, household, the people that they live with, and consumption of supplements and alcohol.

Data were analyzed using the IBM SPSS Statistics 21. Initially, Kolmogorov-Smirnov test was applied to ensure that the data was normally distributed. Chi-square test was performed to determine the relation between gender and general obesity, central obesity, and FMI status. To compare the mean of quantitative variables among binary qualitative variables, Independent Sample T-test was used for normally distributed data and Mann-Whitney test for non-normally distributed ones. To compare the mean of quantitative variables among multinomial qualitative variables, One-way ANOVA was performed for normal distribution and Kruskal-Wallis test for non-normal distribution. If the result of ANOVA was significant, LSD test, and if the Kruskal-Wallis test was significant, Pairwise test were used to do a paired comparison of the groups. In order to assess the correlation between two variables, Pearson’s Product-Moment Correlation and Spearman Correlation were used for normally and non-normally distributed data, respectively. Eventually, all the variables that had significant relationship with body composition indexes entered to the multiple linear regression analysis. A p-value of less than 0.05 was statistically significant.

## 3. Findings

The data of 12 subjects out of 380 ones were excluded due to incomplete information and the analysis was done on the data from 368 participants with the average age of 70.88±7.11 years, including 107 men (29.1%) and 261 women (70.9%). 4.6% of the subjects were underweight, 41% overweight and 19.3% obese. Underweight was more prevalent among men while overweight and obese rates were more common in women. Based on the cut-off of Asian population for waist circumference, 62.2% of subjects and based on the cut-off of NIH, 28.5% of them suffered from central obesity (Table 1).

**Table 1:** Comparison of anthropometric measurements between men and women

Variable	Men n=107 (%)	Women n=261 (%)	Total n=368 (%)	p
**BMI (kg/m^2^)**				
**-Underweight (<20)**	10 (9.3)	7 (2.7)	17 (4.6)	<0.001
**-Norma l(20-24.9)**	56 (52.3)	73 (28)	129 (35.0)	
**-Overweight (25-29.9)**	29 (27.2)	122 (46.7)	151 (41)	
**-Obese (>30)**	12 (11.2)	59 (22.6)	71 (19.3)	
**Waist Circumference (cm)**				
**-Asian Cut-off**				
**Normal**	54 (53.27)	82 (31.4)	136 (39.95)	<0.001
**Obese**	50 (46.73)	179 (8.6)	229 (60.05)	
**-NIH**				
**Normal**	94 (87.9)	169 (64.8)	263 (71.5)	<0.001
**Obese**	13 (12.1)	92 (35.2)	105 (28.5)	
**FMI**				
**-Normal**	36 (33.6)	53 (20.3)	89 (24.2)	0.02
**-High**	55 (51.4)	155 (59.4)	21 (57.1)	
**-Very high**	16 (15.0)	53 (20.3)	69 (18.7)	

In addition, only 24.2% of subjects had normal FMI, while 57.1% of them had high FMI and 18.7% very high FMI. High FMI was found in 47% of men and 37.5% of women with normal BMI (Table 1). In this study, average FM, body fat percentage, and FMI in women were significantly higher than men (p<0.001), whereas FFM and its percentage were significantly higher in men (P<0.001) (Table 2).

**Table 2:** Comparison of body composition indicators between men and women

Variable	Men n=107 (M±SD)	Women n=261 (M±SD)	Total n=368 (M±SD)	p
**BMI (kg/m^2^)**	24.5±3.8	27.0±3.8	26.2±4.03	<0.001
**Body fat mass (kg)**	17.08±5.84	23.3±5.5	21.49±6.27	<0.001
**Fat free mass (kg)**	52.06±6.92	41.26±5.31	44.4±7.61	<0.001
**Body fat percentage (%)**	24.27±4.44	35.76±4.08	32.42±6.69	<0.001
**Fat free percentage (%)**	75.72±4.44	64.24±4.08	67.58±6.69	<0.001
**FMI (kg/m^2^)**	6.08±2.07	9.77±2.34	8.7±2.82	<0.001

Body fat percentage was positively associated with age in both men and women (p<0.001), while fat free percentage, BMI and waist circumference had significant negative relationship with age in both genders (p<0.001) (Table 3).

**Table 3:** Association between body composition indicators and age

Variable	Age (year)					

	Men n=107		Women n=261		Total n=368	

	r	p	r	P	r	p
**Body fat percentage**	0.42	<0.001	0.22	<0.001	0.01	0.90
**Fat free percentage**	-0.42	<0.001	-0.22	<0.001	-0.01	0.90
**FMI (kg/m^2^)**	0.06	0.54	-0.07	0.23	-0.09	0.06
**BMI (kg/m^2^)**	-0.26	0.01	-0.36	<0.001	-0.37	<0.001
**Waist circumference (cm)**	-0.26	0.01	-0.30	<0.001	-0.18	<0.001

While the average daily energy intake (p=0.04) and the percentage of energy from protein (p=0.01) were significantly more in men, women had more percentage of energy from fat (p=0.001). There were no significant difference in the percentage of energy from carbohydrate between men and women (p>0.05). Energy intake and the percentage of energy from fat had significant positive relationship with waist circumference, body fat percentage and FMI and significant negative relationship with fat free percentage (p<0.05) (Table 4).

**Table 4:** Association between energy intake and percentage of energy from macronutrients and anthropometric measurements

Variable	Energy (kcal/day)		Carbohydrate (%)		Protein (%)		Fat (%)	

	r	p	r	p	r	p	r	p
**BMI (kg/m^2^)**	0.29	0.02	-0.07	0.11	-0.05	0.36	0.26	0.02
**Waist circumference (cm)**	0.28	0.02	-0.06	0.13	0.05	0.33	0.20	0.02
**Body fat percentage**	0.2	0.03	-0.05	0.17	-0.1	0.04	0.25	0.02
**Fat free percentage**	-0.2	0.03	0.05	0.17	0.1	0.04	-0.25	0.02
**FMI (kg/m^2^)**	0.22	0.03	-0.03	0.21	-0.11	0.04	0.29	0.02

Moreover, the percentage of energy from protein was significantly negatively associated with body fat percentage and FMI and positively associated with fat free percentage (P<0.05) (Table 4). Average weekly physical activity (cpm), physical activity on weekdays (cpm), the number of steps per day and percentage of moderate to vigorous physical activity (MVPA) were all significantly negatively associated with body fat percentage, FMI and BMI. In contrast, weekly percentage of sedentary activity, percentage of sedentary activity during weekends, weekly percentage of light activity, percentage of light activity in the weekdays and weekends were positively associated with waist circumference. Similarly, there was positive relationship between percentage of light activity in the weekdays and weekends and BMI (Table 5).

**Table 5:** Partial correlation between physical activity and body composition indicators

Variables	Body fat (%)		FMI (kg/m^2^)		BMI (kg/m^2^)		Waist circumference (cm)	

	r	p	r	p	r	p	r	p
**physical activity in the week (cpm)**	-0.48	<0.001	-0.39	0.004	-0.28	0.03	-0.25	0.08
**physical activity on weekdays (cpm)**	-0.48	<0.001	-0.42	0.002	-0.36	0.01	-0.30	0.04
**physical activity on weekends (cpm)**	-0.27	0.053	-0.21	0.14	-0.12	0.41	-0.06	0.70
**The number of steps per day**	-0.48	<0.001	-0.41	0.003	-0.33	0.02	-0.33	0.02
**Sedentary percentage in the week**	0.01	0.94	0.08	0.60	0.19	0.19	0.25	0.08
**Sedentary percentage on weekdays**	0.04	0.80	0.03	0.82	0.12	0.39	0.18	0.20
**Sedentary percentage on the weekend**	0.02	0.89	0.01	0.49	0.20	0.15	0.27	0.05
**Percentage of light activity in the week**	0.08	0.56	0.16	0.25	0.27	0.05	0.34	0.02
**Percentage of light activity on the weekdays**	0.07	0.61	0.14	0.34	0.22	0.12	0.28	0.05
**Percentage of light activity on the weekend**	0.08	0.55	0.17	0.24	0.27	0.06	0.34	0.02
**Percentage of MVPA in the week**	-0.66	<0.001	-0.65	<0.001	-0.64	<0.001	-0.66	<0.001
**Percentage of MVPA on the weekdays**	-0.61	<0.001	-0.60	<0.001	-0.58	<0.001	-0.56	<0.001
**Percentage of MVPA on the weekend**	-0.51	<0.001	-0.52	<0.001	-0.52	<0.001	-0.55	<0.001

r= Partial correlation

In this study, body fat percentage, FMI, BMI and waist circumference were significantly higher in less educated people. FMI and waist circumference, and body fat percentage and fat free percentage were statistically different between all levels of education (P<0.05). Additionally, people with primary school degree had higher BMI than those with secondary school, high school and diploma and university degree. Meanwhile, body fat percentage and FMI in currently working subjects were significantly lower, while body fat percentage, FMI, and BMI in people who were homemaker in the past were significantly higher. The mean difference of body fat percentage and fat free percentage, and FMI and waist circumference were significant in homemakers compared to the employed and self-employed ones. Additionally, there was significant difference between employed and homemaker participants and also between employed and self-employed ones (Table 6).

**Table 6:** Association between socio-demographic factors and anthropometric measurement

Variable	Fat free percentage (M±SD)	Body fat percentage (M±SD)	FMI (M±SD)	BMI (M±SD)	Waist circumference (M±SD)
**Education**					
**-Primary school**	64.32±5.88	35.68±5.88	10.10±2.81	27.87±4.24	89.21±8.62
**-Secondary school, high school and Diploma**	68.08±5.93	31.92±5.93	8.37±2.45	25.83±3.64	86.16±9.08
**-University degree**	71.91±7.58	28.09±7.58	7.21±2.93	24.87±4.04	87.29±7.13
**p-value**	<0.001	<0.001	<0.001	<0.001	0.01
**Currently occupied:**					
**-No**	66.98±6.27	33.02±6.27	8.88±2.73	26.39±4.02	87.02±8.83
**-Yes**	72.84±7.92	27.16±7.92	7.11±3.12	25.40±4.06	89.35±7.60
**p value**	<0.001	<0.001	<0.001	0.80	0.30
**Former job**					
**-Unemployed/homemaker**	63.51±3.82	36.49±3.82	10.11±2.22	27.45±3.68	86.21±7.52
**-Self-employed**	70.31±6.37	29.69±6.37	7.99±2.78	26.32±4.14	91.24±9.44
**-Employee**	71.5±6.79	28.5±6.79	7.21±2.65	24.72±3.92	86.48±9.27
**p value**	<0.001	<0.001	<0.001	<0.001	<0.001
**Time on TV**					
**-<2 hours**	70.95±6.27	29.05±6.27	6.88±1.90	23.47±2.48	83.16±7.75
**->2 hours**	64.49±5.47	35.51±5.47	10.36±2.48	28.87±3.40	91.02±7.86
**p value**	<0.001	<0.001	<0.001	<0.001	0.67
**Chronic disease**					
**-No**	70.75±6.68	29.24±6.68	7.36±2.46	24.69±3.37	85.43±7.99
**-Yes**	65.45±5.81	34.55±5.81	9.60±2.69	27.36±4.10	88.49±9.01
**p value**	<0.001	<0.001	<0.001	0.003	0.23
**Medication**					
**-No**	70.13±6.75	29.87±6.75	7.57±2.47	24.90±3.35	85.89±8.10
**-Yes**	65.77±6.04	34.23±6.04	9.50±2.79	27.28±4.19	88.24±9.05
**p value**	<0.001	<0.001	<0.001	0.003	0.22
**Smoking**					
**-No**	66.68±6.52	33.47±6.27	9.06±2.76	26.47±4.05	86.65±8.40
**-Yes**	72.91±5.07	26.15±5.61	6.57±2.15	25.18±3.76	90.88±9.81
**p value**	<0.001	<0.001	<0.001	0.21	0.48
**Salary (monthly)**					
**-< 300$**	66.52±5.23	33.48±5.23	9.14±2.56	27.37±4.04	86.95±9.15
**-> 300$**	67.27±6.81	32.73±6.81	8.57±2.76	25.69±3.91	87.43±8.51
**p value**	0.19	0.19	0.23	0.65	0.58
**Home possession**					
**-Rental/mortgage**	67.84±5.92	32.16±5.92	9.09±2.67	25.88±3.98	86.72±8.65
**-Owner**	68.24±6.85	31.76±6.85	8.45±2.82	27.36±3.99	88.68±8.84
**p value**	0.31	0.31	0.11	0.80	0.58
**Marital status**					
**-Single/alone**	66.68±6.16	33.32±6.16	9.22±2.88	26.37±4.34	86.36±8.64
**-Married**	67.26±6.75	32.74±6.75	8.82±2.78	26.26±3.93	87.58±8.76
**p value**	0.37	0.37	0.18	0.34	0.57
**Household**					
**-Alone**	66.21±6.2	33.79±6.20	9.51±3.06	26.80±4.72	87.10±9.18
**-Spouse, spouse and children**	67.19±6.71	32.81±6.71	8.54±2.77	26.28±3.92	87.59±8.79
**-Children, other relatives**	66.51±6.51	33.49±6.51	8.84±2.79	25.92±4.05	85.84±8.09
**p value**	0.21	0.21	0.10	0.55	0.59
**Ethnic origin**					
**-Persian**	67.61±6.31	32.39±6.3	8.61±2.68	26.10±4.00	86.56±8.98
**-Other**	67.54±7.25	32.46±7.25	8.83±3.02	26.57±4.08	88.31±8.27
**p value**	0.54	0.54	0.46	0.64	0.34
**Supplement**					
**-No**	67.81±7.16	32.19±7.16	8.73±3.07	26.44±4.29	88.58±8.05
**-Yes**	67.35±6.20	32.64±6.2	8.66±2.56	26.13±3.76	85.93±9.20
**p value**	0.98	0.98	0.81	0.06	0.06
**Alcohol**					
**-No**	67.5±6.72	32.5±6.72	8.71±2.84	26.27±4.05	87.05±8.75
**-Yes**	69.55±5.83	30.45±5.83	8.29±2.46	26.81±3.58	92.50±6.47
**p value**	0.21	0.21	0.58	0.69	0.13

In the current study, body fat percentage, FMI and BMI in people who watched TV more than 2 hours per day were significantly higher. In addition, body fat percentage, FMI and BMI in those who suffered from illnesses and took medicine were significantly higher. Meanwhile, non-smokers had higher body fat percentage and FMI compared to the smokers (Table 6).

The results of multiple linear regression showed that older ages, being female, more hours of watching TV, suffering from diseases, higher energy intake, higher percentage of energy from fat and lower levels of physical activity were contributed to the higher anthropometric measurements including BMI, FMI, waist circumference and body fat percentage (Table 7).

**Table 7:** Factors contributing to the anthropometric measurements

No	Model	Predictor variable	R^2^	Adjusted R^2^	Beta	t	p
**1**	**BMI**	Age	0.54	0.49	0.11	2.56	0.01
		Gender			0.26	4.64	<0.001
		Time on TV			0.38	7.26	<0.001
		Diseases			0.11	2.29	0.02
		Energy intake			0.59	11.24	<0.001
		Physical activity			-0.15	-2.81	0.01
**2**	**Waist circumference**	Age	0.38	0.35	0.18	5.3	<0.001
		Gender			0.54	13.66	<0.001
		Energy intake			0.24	5.34	<0.001
		Percentage of fat intake			0.45	9.84	<0.001
**3**	**Body fat percentage**	Age	0.51	0.46	0.21	5.53	<0.001
		Gender			0.73	13.87	<0.001
		Time on TV			0.11	2.29	0.02
		Energy intake			0.25	5.41	<0.001
		Percentage of fat intake			0.15	2.93	0.01
		Physical activity			-0.1	-2.94	0.01
**4**	**FMI**	Gender	0.45	0.41	0.53	9.05	<0.001
		Energy intake			0.41	7.79	<0.001
		Percentage of fat intake			0.15	2.62	0.01
		Physical activity			-0.22	-4.24	<0.001

## 4. Discussion

The results of this study showed that overweight and obesity were common among the elderly people. 41% and 20% of all the subjects were overweight and obese, respectively, and they were more female than male. These statistics were similar to the previous studies. For instance, in a study among the adults of district 13 of Tehran, Iran the prevalence of overweight and obesity was 63% and those problems were more common in women than in men (Azadbakht, Mirmiran, & Azizi, 2001). In addition, findings in Isfahan, Iran showed that 39% of men were overweight and 12% were obese while 28.9% of women were obese (Alavi-Naeini, Dorosty, & Aghdak, 2003). In contract, the prevalence of overweight and obesity among the aged people in Khorasan Razavi, Iran were 28.9% and 11.7%, respectively although obesity in women was more than in men (Nematy et al., 2009). The lower prevalence in the last study may be due to the fact that they included both urban and rural people, while in current study only urban elderly was assessed. Interestingly, in Tunisia the prevalence of obesity in women (21.7%) was more than in men (9.4%) (Hammami, Barhoumi, Kammoun, Turki, & Hajem, 2009). However, in the Spanish elderly, the prevalence of overweight and obesity in men were 49% and 31.5%, respectively, while in women were 39.8% and 40.8%, respectively (Gutierrez-Fisac, Lopez, Banegas, Graciani, & Rodriguez-Artalejo, 2004). Other studies also showed more prevalence of obesity in women (Fidanza, Coli, Fiorucci, Coli, & Sarchielli, 1990; Perissinotto, Pisent, Sergi, Grigoletto, & Enzi, 2002). The higher prevalence of obesity among women may be caused by multiple childbirths, hormonal and physiologic and metabolic differences and less physical activity.

In this study, central obesity was around 28% (12.1% men and 35.2% women) based on the cut-off of NIH and 62.2% (46.7% men and 68.6% women) based on the cut-off of Asian populations. Previous studies reported similar prevalence based on the cut-off of NIH. For example, the prevalence of central obesity among the elderly of Khorasan Razavi, Iran was 18.6% and 63.1% in men and women, respectively (Nematy et al., 2009). Furthermore, it was 32.1% and 69.7% among Portuguese men and women, respectively (Sardinha et al., 2012). Moreover, 48.4% of Spanish old men and 78.4% of Spanish old women have suffered from central obesity (Gutierrez-Fisac et al., 2004). Meanwhile, in Saudi Arabia it was 34.4% (Abolfotouh, Daffallah, Khan, Khattab, & Abdulmoneim, 2001).

In this study, the average body fat percentage of participants was 32.42% and women had higher body fat percentage than men. Also, the FMI of more than 57% of total participants was high and about 19% very high. The results of current study confirmed the previous researches. For instance, the average body fat percentage of the elderly was 28.97% in the governmental care homes for the aged people in Kermanshah, Iran (Pasdar et al., 2011). Additionally, a recent study on the Canadian elderly showed that the body fat percentage was 26.5% in men and 40.2% in women (McIntosh, Smale, & Vallis, 2013). Moreover, in Amsterdam, 74% of elderly people had high and very high FMI (Konijn et al., 2014).

In this study, there was significant positive relation between body fat percentage and age in both genders, while BMI and waist circumference were negatively associated with age. The results of current study were in line with previous studies. For example, in the study by Atlantis et al. on the men aged 35-81 years, body fat percentage increased by age while body muscular mass decreased and after the age of 60 the waist circumference either did not change or decreased by age (Atlantis, Martin, Haren, Taylor, & Wittert, 2008). Results of a study in a period of 9 years showed that the body fat tissue in both male and female elderly decreased. Moreover, people over 75 lost more FFM in comparison with people between 65 and 74 years (Genton et al., 2011). In the current study, FMI decreased by age only in women. The study by Hughes et al. showed that body fat percentage of women was more than men and the body fat mass had increased by age but after the age of 70 it decreased in women (Hughes, Frontera, Roubenoff, Evans, & Singh, 2002). In addition, Baumgartner et al. showed that FFM decreased by age in both genders and fat mass decreased in women (Baumgartner, Stauber, McHugh, Koehler, & Garry, 1995). In this study, FMI decreased in women, whereas increased in men by age. In other studies on the elderly, BMI and FMI similarly decreased by age (Alavi-Naeini et al., 2003; Elia, 2001; McIntosh et al., 2013; Perissinotto et al., 2002).

The results of this study showed that energy intake and the percentage of energy from fat were positively associated with waist circumference, BMI, body fat percentage and FMI. In the study by Howarth et al. higher BMI had relationship with higher energy intake and percentage of energy from fat (Howarth, Huang, Roberts, Lin, & McCrory, 2006). In addition, result of a study among elderly people aged 65 or more and living in long term care institutions showed that daily fat intake had significant association with waist circumference, mid-arm circumference and BMI (Rodrigues et al., 2012). Meanwhile, Miller et al. (1990) found that percentage of body fat increased with the increased percentage of energy from fat (Miller, Lindeman, Wallace, & Niederpruem, 1990). In the current study, the percentage of energy from protein was positively associated with fat free percentage. Similarly, in the previous studies, positive relationship between protein intake and the decrease in body muscular mass loss was found (Drewnowski & Greenwood, 1983; Loenneke & Pujol, 2011; Miller et al., 1990).

The results of this study indicated that there was significant negative relationship between physical activity and body fat percentage, FMI and BMI. The probable mechanism is that when the physical activity decreases, if the resulting decrease in the energy consumption is unparalleled with energy intake, it leads to increasing weight and FM, and subsequently obesity. Other studies showed negative association between physical activity and body fat percentage in the elderly (Chastin, Ferriolli, Stephens, Fearon, & Greig, 2012; Gaba et al., 2010; Whitt, Kumanyika, & Bellamy, 2003).

In this study, education was negatively related to body fat percentage, FMI, BMI and waist circumference and positively related to fat free percentage. People with higher levels of education may have healthier lifestyle, including more physical activity, which leads to better control of body composition. Other studies also reported negative association between education and BMI (Alavi-Naeini et al., 2003; Atlantis et al., 2008; Azadbakht et al., 2001; Dey, Rothenberg, Sundh, Bosaeus, & Steen, 2001; Sotoudeh, Khosravi, Khajehnasiri, & Khalkhali, 2005; Young, 1996).

The homemaker participants had significantly more body fat percentage and BMI, and less fat free percentage, probably because in this study, homemakers were all women, and as the results showed, women generally had higher body fat percentage than men. Similarly, a study of the elderly of Isfahan, Iran showed that the BMI of homemakers was higher than those of other professions (Alavi-Naeini et al., 2003). Additionally, the study by Mason et.al among the Finish elderly showed that those who were jobless had higher BMI (Lahti-Koski, Pietinen, Heliövaara, & Vartiainen, 2002).

In this study, body fat percentage, FMI and BMI were significantly higher in the participants who watched TV for more than 2 hours per day contributing to reduce physical activity. This decline in physical activity increased the incidence of obesity. Previous studies similarly confirmed the positive relationship between watching TV and obesity among the Iranian elderly (Alavi-Naeini et al., 2003) and Spanish adults (Vioque, Torres, & Quiles, 2000).

Non-smokers had higher body fat percentage and FMI and less fat free percentage compared to the smokers. The study by Alavi-Naeini et al. on the elderly of Isfahan, Iran showed that smokers had less BMI (Alavi-Naeini et al., 2003). Additionally, a study among Australian adults reported that body fat percentage of smokers is less than non-smokers (Atlantis et al., 2008). Body fat percentage, FMI and BMI were significantly higher in the participants suffered from chronic illnesses. In the previous studies, chronic illnesses had positive relationship with muscular mass loss in the elderly people (Koster et al., 2010; Park et al., 2007).

The results of multiple linear regression showed that age, gender, TV watching time, illnesses, energy intake, percentage of energy from fat and physical activity were contributed to the status of BMI, waist circumference, FMI and body fat percentage. Similarly, Hughes et al. reported that age, physical activity and gender were contributed to the body fat percentage (Hughes et al., 2002). Additionally, Atlantis et al. in the Florey Adelaide Male Aging Study on 1200 men using logistic regression found that ethnicity, older age, smoking, higher energy intake and percentage of energy from carbohydrate were the risk factors of higher body fat percentage, whereas alcohol drinking, physical activity and percentage of energy from fat and protein did not have significant contribution toward the indicators of body composition (Atlantis et al., 2008).

## 5. Conclusion

The current study showed that overweight, obesity and high body fat percentage, which were remarkable risk factors of non-communicable diseases, were common among the elderly of district 5 of Tehran, Iran. In addition, anthropometric measurements were significantly associated with gender, age, physical activity, energy intake, percentage of macronutrients, educational level, job status, TV watching time, smoking, diseases, and taking medicine. Moreover, older ages, female gender, more TV watching time, diseases, higher energy intake, higher percentage of fat intake, and lower physical activity were contributed to higher BMI, waist circumference, body fat percentage and FMI. To enhance the health condition of the elderly it is recommended that beside increasing their awareness about nutritional balance and variety, they must be encouraged to have more physical activity. Further research is necessary.
